# Clinical and electrophysiological evaluation of carpal tunnel syndrome: approach and pitfalls

**DOI:** 10.17712/nsj.2017.3.20160638

**Published:** 2017-07

**Authors:** Mohammed H. Alanazy

**Affiliations:** *From the Division of Neurology, Department of Internal Medicine, King Saud University Medical City, King Saud University, Riyadh, Kingdom of Saudi Arabia*

## Abstract

One of the most common referrals to the electrodiagnostic (EDX) laboratory is to confirm a clinical impression of carpal tunnel syndrome (CTS). The EDX studies are valuable in localizing median nerve abnormalities to the wrist, grading its severity, and excluding other condition that can mimic or coexist with CTS. However, there are many clinical and EDX pitfalls that can lead to misdiagnosis. Careful clinical assessment and attention to technical factors and details of the EDX techniques are fundamental for the quality and accurate interpretation of the study. This review aims to discuss the clinical and the EDX approaches to the diagnosis of CTS with emphasis on the commonly encountered pitfalls.

Carpal Tunnel Syndrome (CTS) is a common medical problem caused by compression of the median nerve as it passes beneath the transverse carpal ligament at the wrist. It is the most common entrapment neuropathy encountered in the electrodiagnostic (EDX) laboratory.^1^ In one study, the reported incidence of CTS was 376 per 100,000 person -years and women were affected twice as common as men.^2^ The reported prevalence of clinically and electro-physiologically confirmed CTS was 2.7%.^3^ Confirmation of the clinical diagnosis of CTS is achieved by the EDX studies, including nerve conduction study (NCS) and electromyography (EMG). The EDX studies are valuable in localizing median nerve abnormalities to the wrist, grading its severity, and excluding other condition that can mimic or coexist with CTS. Accurate interpretation of the EDX studies requires the electromyographer to have the knowledge and experience of recognizing EDX pitfalls that may arise when conducting the procedure. In this article, we review the clinical and EDX approach to diagnose CTS with emphasis on the typical patterns of EDX abnormalities, and describe the common EDX pitfalls.

## Clinical approach

In approaching a patient with hand symptoms, physicians need to consider disorders affecting the central and peripheral nervous system in the differential diagnosis. The latter includes disorders of motor neurons, nerve roots, brachial plexus, peripheral nerves, neuromuscular junctions, and muscles.

## Clinical features of CTS

The defining symptoms of CTS include intermittent paresthesia, with or without pain, involving the lateral 3 ½ digits that often awaken patients from sleep. Pain and paresthesia proximal to the wrist level have been reported in 20-40% of CTS patients.^4^ Subjective feeling of paresthesia involving the whole hand may occur,^5^ however, objective sensory signs remain confined to the median-nerve territory. Carpal Tunnel Syndrome symptoms are typically provoked by sustained or repetitive hand activities such as holding a book, typing etc., and relieved by shaking the hands out, placing them under running water or using a splint. When CTS is left untreated, paresthesia may become constant with subsequent development of weakness and atrophy of median-innervated thenar muscles. The symptoms may improve or resolve with modification of hand activities, but future recurrence remains possible.

As for the neurological examination, the physician should identify all muscles with weakness and distribution of sensory impairment, and integrate these findings to determine lesion localization. In CTS, neurological signs are limited to median-nerve territories distal to the carpal tunnel. Neurological examination may be normal particularly in mild CTS. Sensory impairment usually involves palmar surface of the lateral 3 ½ fingers and spares thenar eminence; the palmar cutaneous sensory branch of the thenar eminence arises proximal to the carpal tunnel. Sensory loss of the thenar eminence suggests a proximal localization. Muscle-strength assessment may reveal weakness in abductor pollicis brevis (APB) and opponens pollicis (OP). Atrophy of the thenar muscles is seen at a more severe stage when axonal loss has developed. Provocative maneuvers such as Tinel and Phalen tests are not usually helpful as they lack high sensitivity and specificity.^4^

## Clinical features not suggestive of CTS

Physicians should be aware of the clinical features that are not consistent with CTS, not only to avoid misdiagnosis but also to provide the appropriate management for the underlying condition. When abrupt-onset pain and weakness are the presenting features, vasculitic neuropathy, Parsonage-Turner syndrome, trauma, and spontaneous tendon rupture are to be considered in the differential diagnosis. Neck pain provoked by neck movement and radiated to the upper limb favors a diagnosis of cervical radiculopathy. Shoulder pain provoked by arm movement favors the presence of a shoulder problem (e.g., adhesive capsulitis, rotator cuff disorders). Disorders of carpometacarpal joint (CMJ) of the thumb (e.g. osteoarthritis), and thumb extensor tendons (e.g. tendinitis) are common causes of hand pain that are recognized clinically by demonstrating tenderness over the first CMJ and thumb extensor tendons, respectively. Disorders of the annular ligaments of the fingers (A pulley) can cause digit pain and locking during finger flexion-extension movement, tenderness and palpable snapping or crepitus over the A pulley. Morning stiffness of the small joints of the hands and metacarpophalangeal (MCP) joint tenderness and swelling are features suggestive of rheumatoid arthritis.

Regarding clinical signs, sensory loss that involves all fingers can be due to combination of median and ulnar neuropathies, polyneuropathy, brachial plexopathy, cervical polyradiculopathy, sensory neuronopathy, myelopathy, thalamic or cortical lesions. When CTS coexists with a length-dependent polyneuropathy, the glove-stocking sensory loss of peripheral neuropathy is generally worse on the lateral 3 ½ digits than the medial 1 ½ digits. Painless hand weakness with normal sensory examination is not a feature of CTS, rather one should consider multifocal motor neuropathy with/without conduction block (MMN), motor neuron disease (MND) such as progressive muscular atrophy (PMA), distal myopathies, or a cortical lesion located in the hand motor area. In MMN, weakness is confined to the muscles innervated by the affected nerve distal to the site of conduction block. In PMA, muscle weakness occurs in a myotomal distribution, where all muscles innervated by the involved spinal cord segment will manifest clinical weakness and/or EMG evidence of denervation. In inclusion body myositis (IBM), weakness preferentially involves long finger flexors and quadriceps. In distal muscular dystrophies, weakness variably involves finger extensors and distal leg muscles depending on its subtype.

## Localization pitfalls

Fascicular involvement of a proximal nerve segment may falsely suggest a more distal site of localization than the actual site of the lesion.^6^ This is because muscles supplied by the spared nerve fascicle remain strong despite being located distal to the lesion. Thus, the site of the lesion can best be localized at or proximal to the most proximal involved muscle, best identified by needle EMG to capture subclinical muscle involvement. Clinically, isolated weakness of APB and OP, when all other muscles are normal, suggests a median nerve lesion at or proximal to the carpal tunnel. When weakness is restricted to flexor pollicis longus (FPL), flexor digitorum profundus (FDP)-2 & 3 and pronator quadratus (PQ), a compressive lesion of the anterior interosseous nerve (AIN), or a fascicular lesion of the median nerve trunk in the upper arm should be excluded.^7^ More details about abnormal muscles associated with different lesion sites are described in **Table 1**.

**Table 1:** Sensory motor NCS and needle EMG findings in disorders that may mimic Carpal Tunnel Syndrome.

Disorders	Sensory NCS	Motor NCS	Muscles involved by EMG
Motor neuron disease e.g. ALS	Normal^38^	Median CMAP: low amplitude Ulnar CMAP: low amplitude^38^	EMG signs of LMN dysfunction in at least 2 of the 4 CNS regions: bulbar, cervical, thoracic, or lumbosacral spinal segments^38^
C8/T1 radiculopathy	Normal^39^	Median CMAP: normal or low amplitude Ulnar CMAP: normal or low amplitude^39^	All or some of C8/T1 supplied muscles (APB, FDI, ADM, FPL, EIP, and paraspinals)^40^
Thoracic outlet syndrome (lower trunk)	Median SNAP: normal Ulnar and medial antebrachial SNAP: low amplitude^41^	Median CMAP: low amplitude Ulnar CMAP: less severe involvement than median CMAP^42^	All or some of C8/T1 supplied muscles (T1 worse than C8), sparing paraspinals^41^
Medial cord lesion	Median SNAP: normal Ulnar and medial antebrachial SNAPs: low amplitude^41^	Median CMAP: low amplitude Ulnar CMAP: low amplitude^41^	C8/T1 muscles; spares fibers traveling through posterior cord (e.g., EIP) and Paraspinals^41^
C5 radiculopathy	Normal^39^	Median CMAP: normal Ulnar CMAP: normal^39^	C5 muscles: supraspinatus, infraspinatus, deltoid, brachioradialis, biceps, and C5 paraspinals^40^
C6 radiculopathy	Normal^39^	Median CMAP: normal Ulnar CMAP: normal^39^	C6 muscles: as in C5 + PT, FCR, triceps, anconeus, EDC and C6 paraspinals^40^
C7 radiculopathy	Normal^39^	Median CMAP: normal Ulnar CMAP: normal^39^	C7 muscles: triceps, anconeus, PT, FCR, EDC, and C7 paraspinals^40^
Upper trunk	Median-D1& 2 SNAP: low amplitude Radial SNAP: low amplitude Lateral antebrachial: low amplitude Ulnar SNAP: normal^41^	Median CMAP: normal Ulnar CMAP: normal^41^	All or some of C5/6 muscles (listed above) sparing paraspinals, serratus anterior and rhomboids^41^
Lateral cord	Median-D1, 2 & 3 SNAP: low amplitude Lateral antebrachial: low amplitude Ulnar SNAP: normal^41^ Radial SNAP: normal^41^	Median CMAP: normal Ulnar CMAP: normal^41^	Biceps, brachialis, PT, and FCR^41^
Median nerve at or proximal to the elbow	Median SNAP: decreased Ulnar SNAP normal^43^	Median CMAP: low amplitude Ulnar CMAP: normal^43^	APB, FPL, FDP-D2 & 3, FDS, PQ, FCR and PT^43^
AIN neuropathy	Normal	Normal	FPL, FDP-D2 & 3, PQ^43^
Length dependent axonal polyneuropathy	Sural SNAPs are affected earlier and more severely than upper limb SNAPs^44^	Lower limb CMAPs are affected earlier and more severely than upper limb CMAPs^44^	Denervation is worse in distal compared to proximal muscles, and in lower more than upper limbs^44^

ADM - abductor digiti minimi, AIN - anterior interosseous nerve, ALS - amyotrophic lateral sclerosis, APB - abductor pollicis brevis, CMAP - compound muscle action potential, EDC - extensor digitorum communis, EIP - extensor indicis proprius, EMG - electromyography, FCR - flexor carpi radialis,

FDI - first dorsal interosseous, FDP D2 & 3 - flexor digitorum profundus digits 2 and 3, FDS - flexor digitorum superficialis, FPL - flexor pollicis longus, LMN - lower motor neuron, NCS - nerve conduction study, PQ - pronator quadratus, PT - pronator teres, SNAP - sensory nerve action potential

## The EDX studies

Carpal Tunnel Syndrome is a clinical diagnosis based on the presence of the typical symptoms described above. There is no single gold standard test for the diagnosis of CTS. The NCS are valid and reliable in confirming the clinical impression of CTS with a sensitivity of >85% and a specificity of 95%.^8^ The value of NCS is to confirm the clinical diagnosis of CTS, assess its severity and rule in/out coexistent conditions. Performance of an accurate NCS requires using proper techniques with recognition and correction of the pitfalls that may arise during the procedure. Because many patients with CTS have abnormalities discernable only by relative differences in latencies on comparison studies, careful consideration of physiologic and technical factors is crucial to ascertain validity of the results. Errors with electrodes placement, distance measurement, stimulation site, cathode-anode orientation, stimulus intensity, and filter sitting, must be identified and rectified during the procedure, as these errors are not usually recognizable during data interpretation. Failure to capture electrodiagnostic abnormalities especially in mild CTS (false-negative) is less hazardous than the false diagnosis of a normal subject with CTS (false-positive). The latter may influence treatment decisions and subject the patient to an unwarranted intervention. Therefore, a minimum of 2 comparative tests demonstrating relatively prolonged median latencies are required to minimize a false-positive result.^9^ On the other hand, NCS may show incidental median nerve slowing at the wrist in asymptomatic workers, diabetics, or in the context of demyelinating neuropathies. Therefore, when median nerve slowing at the wrist is identified, the study should be reported as “median neuropathy at the wrist” rather than “CTS”. The clinical-electrophysiologic correlation is crucial to help clarify whether the NCS finding of “median neuropathy at the wrist” is incidental or consistent with a clinical impression of CTS, and whether a superimposed condition exists or not.

Approximately 10-15% of patients with a clinically defined CTS have normal NCS.^10^ This could occur as a result of intermittent median nerve compression at the wrist that has not caused demyelination or axonal loss to be detected by EDX studies. The other reason is that the range of normal values is wide, and a distal latency value that falls within the “population range of normal values” could have been considered abnormal for an individual if it had been compared with a baseline study before the onset of CTS. The commonly used reference values pertinent to this review are shown in **Table 2**.^11^

**Table 2:** Reference values for the nerve conduction studies used in the evaluation of Carpal Tunnel Syndrome.

Study	Onset-to-peak amplitude: LLN (3^rd^ percentile)	Peak latency: ULN (97^th^ percentile) (ms)	Onset latency: ULN (97^th^ percentile) (ms)	Conduction velocity: LLN (3^rd^ percentile) (m/s)
Digit 2 median antidromic sensory	11 mv*	4.0	3.3	NA
Digit 5 ulnar antidromic sensory	10 mv*	4.0	3.1	NA
Digit 2 median vs digit 5 ulnar peak latency difference	NA	<0.5	NA	NA
Digit 4 median vs digit 4 ulnar peak latency difference	NA	<0.5	NA	NA
Palmar orthodromic peak latency difference.	NA	<0.3	NA	NA
Median motor	4.1 mv*	NA	4.5	49
Ulnar mot	7.9 mv*	NA	3.7	52 (below elbow) 43 (across elbow) 50 (above elbow)

LLN - lower limit normal, ULN - upper limit normal, NA - not applicable.

* Values represent all ages. For stratification by age and body mass index, we refer the reader to the paper of Chen et al^11^

Age, height and weight: Patients’ age, height and weight must be taken into consideration when interpreting NCS data.^9^ The effect of age is particularly manifest at extremes of age. Amplitudes of motor and sensory potentials and Conduction Velocity (CV) decrease with age particularly for patients above 60 years. Individuals with extreme height have a slightly slower CV and lower amplitudes, usually more prominent in the lower than upper limbs. Several studies have reported an association between obesity and median neuropathy at the wrist in asymptomatic subjects.^12^–^14^ Therefore, the electromyographer should avoid overdiagnosing CTS based upon minor abnormalities in NCS parameters that are fully explained by patients’ demographics.

## Temperature Effect

Before starting NCS, cold hands should be sufficiently warmed with an appropriate method to achieve a surface temperature ≥32°C. Cold-hand temperature induces CV slowing and latency prolongation, and an increment in the amplitude and duration of motor as well as sensory potentials.^15^ Previous studies showed that it might not be necessary to control hand temperature when comparison studies are conducted to evaluate for CTS.^16^,^17^ However, controlling hand temperature at ≥32°C was a consensus recommendation by the CTS quality group based on the fact that the comparison studies, recommended by the American Association of Neuromuscular & Electrodiagnostic Medicine (AANEM), were all performed with careful attention to hand temperature.^18^

The presence of prolonged median motor and sensory latencies in a cold hand may create a false diagnosis of “median neuropathy at the wrist”. Any sensory study that shows high-amplitude long-duration sensory nerve action potential (SNAP) along with slow CV should alert the electromyographer to a possible cool-temperature effect.

## Pitfalls with sites and techniques of stimulation and recording: Median Motor NCS

**Figure 1:**
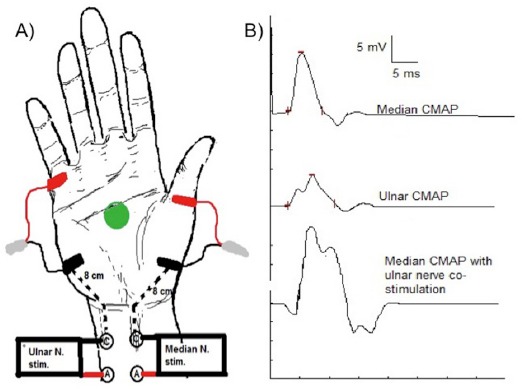
Median and ulnar motor nerve conduction studies recording abductor pollicis brevis and abductor digiti minimi, respectively. **A)** Electrode placement (black: active, red: reference, and green: ground) and stimulation sites. **B)** Compound muscle action potential waveform of the median (top trace) and ulnar (middle trace) nerves. Notice the change in the median waveform when ulnar co-stimulation occurs (bottom trance).

The active electrode (A, black) is placed over the APB muscle belly at the midpoint between the distal wrist crease and the MCP joint of the thumb. The reference electrode (R, red) is placed over the APB tendon at the first MCP joint. The ground electrode (G, green) is placed on the palm or dorsum of the hand. The median nerve is stimulated at the wrist between the tendons of Flexor Carpi Radialis (FCR) and Palmaris Longus, 8 cm proximal to the A electrode. The distance is measured in a hockey-stick-shaped line from the A electrode to the midpoint of the distal wrist crease and then to the point of stimulation following the anatomic course of median nerve (**Figure 1A**). Proximal stimulation is delivered at the antecubital fossa over the brachial artery pulse. At both stimulation sites, the cathode (black) is placed at the point of stimulation closest to the recording electrode (black to black) while the anode (red) is proximal taking care to measure the forearm distance between the stimulation sites (cathodes).

One of the important principles of NCS is to start stimulation at 0 mV (stimulus duration 0.1 ms, low-frequency filter 2-3 Hz, and high frequency filter 10 kHz) with a 3-5 mV increment until the first deflection of baseline appears, noting the point, while the stimulus intensity is kept constant, the cathode is slightly repositioned from side-to-side (sliding technique) looking for the highest potential amplitude to identify the accurate location of the nerve. Sometimes stimulating the median nerve just lateral to the FCR tendon yields higher potential amplitudes with less stimulus intensity. This is followed by moving the A electrode slightly over different points of the APB belly looking for the highest Compound Muscle Action Potential (CMAP) amplitude with a sharp negative deflection from the baseline, which indicates recording from the motor endplate. Once the optimal recording and stimulation sites have been determined, the distance should be readjusted. With every stimulation increment, the CMAP amplitudes grow until no further increment is noticed. Then, a 20% current increment is delivered to ensure stimulation of all axons within the nerve (supramaximal stimulation). If a supramaximal response is not achieved with the maximum current intensity, pulse duration should be increased to the next level. The waveform is carefully observed to ensure that its amplitude and morphology do not change despite the final increment in the stimulus intensity. Application of these techniques guarantees using the lowest possible stimulus intensity to achieve supramaximal stimulation and allows patients to adapt to it. In an average-sized hand, the supramaximal stimulation of median nerve is usually achieved at 30-50 mV.

Failure to determine the appropriate median nerve stimulation site leads to the requirement of higher stimulus intensity. This makes the procedure intolerable and may cause co-stimulation of the ulnar nerve. Improper placement of the A electrode is recognized by the following changes in the median waveform: 1) an initial positive deflection from the baseline, 2) an abnormal waveform morphology and 3) an unexpectedly low CMAP amplitude. An inadvertently reversed cathode-anode orientation (anode distal, cathode proximal) produces a slightly delayed latency because of the longer distance required for action potential propagation from the cathode to the A electrode.

## Sensory NCS

The A electrode is placed over the proximal phalanx of the digit being studied (thumb [D1°, index [D2], middle [D3], ring [D4], and little [D5] fingers) and the R electrode is placed 4 cm distal to the A electrode. The median and ulnar nerves are stimulated at the wrist, 14 cm proximal to their respective A electrodes. The ulnar nerve is routinely stimulated just lateral to the tendon of the flexor carpi ulnaris, but a response can sometimes be obtained by stimulating medial to the tendon. Sensory nerve action potential recorded on the thumb are provoked by stimulating the median and radial nerves at a 10-cm distance from the A electrode. The radial nerve is stimulated on the lateral border of the radius. The median nerve stimulation site and the G electrode placement are as mentioned above. The identical stimulation distance allows internal comparison between the median and each of the ulnar and radial nerves. The stimulus duration used for sensory studies is 0.05 ms, and the low-frequency filter setting is kept at 20 Hz and high-frequency filter setting is kept at 2 kHz.

## Comparison studies

**Figure 2:**
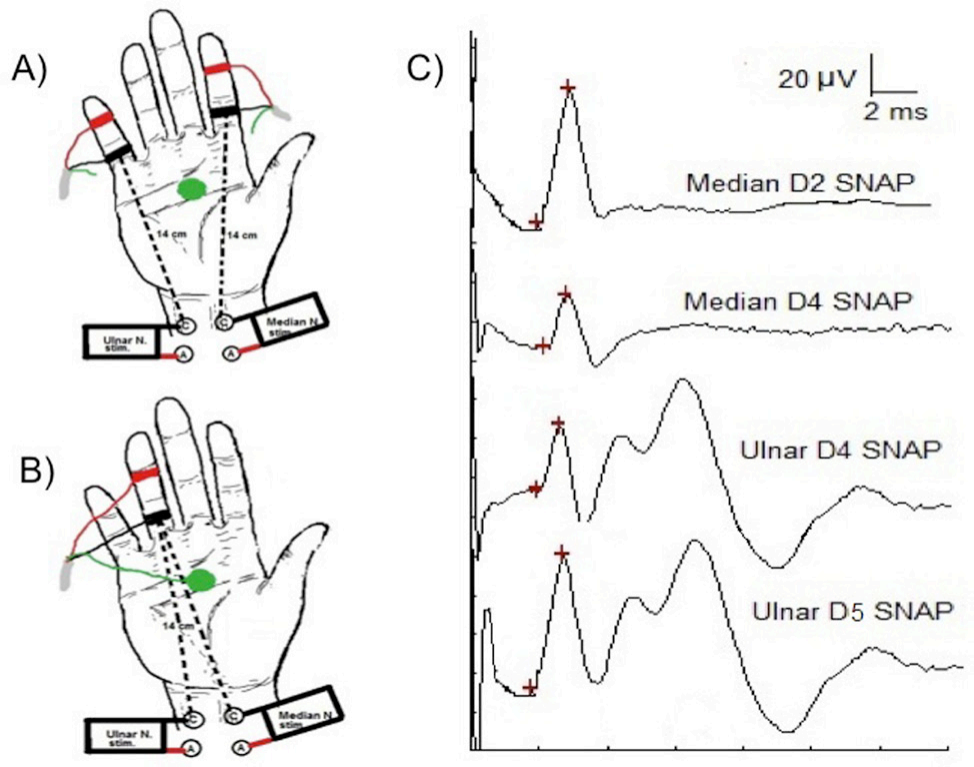
Median-ulnar antidromic sensory comparison study. Electrodes placement (black: active, red: reference, and green: ground) and stimulation sites are shown. **A)** Median digit 2 versus ulnar digit 5. **B)** Median digit 4 vs ulnar digit 4 sensory comparison studies. **C)** Sensory nerve action potential waveform morphology.

**Figure 3:**
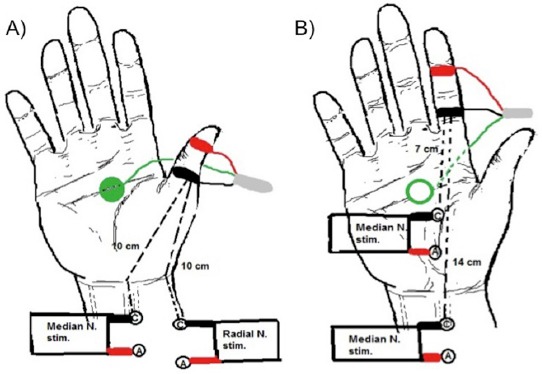
Comparison studies **A)** Median-radial antidromic sensory comparison study. **B)** Segmental median sensory conduction velocity of wrist-to-palm compared to palm-to-digit segments. Electrodes placement (black: active, red: reference, and green: ground) and stimulation sites are shown.

Multiple comparison studies can be used to demonstrate slowing of the median nerve relative to the ulnar or radial nerves. Comparison studies are of paramount importance in providing internal control for confounding factors including age, gender, weight, height, hand size and temperature, as well as the presence of a coexistent polyneuropathy. With such internal control, the only uncontrolled factor is the fact that the median nerve is the only nerve that passes through the carpal tunnel, rendering a relative slowing of its CV/latency attributed to median neuropathy at the wrist. At least 2 abnormal comparison studies are required to lower the risk of a false positive.^9^ These comparison studies include the measurement of the antidromic sensory-latency difference between 1) median D2 and ulnar D5 (**Figure 2A**); 2) median and ulnar D4 (ringdiff) (**Figure 2B**), and 3) median and radial D1 (thumbdiff) (**Figure 3A**). A peak-latency (PL) difference between the median and ulnar or radial nerves of ≥ 0.5 ms is considered significant.^19^

Thumbdiff study is a useful technique to demonstrate relative median-nerve slowing at the wrist when median-ulnar comparison studies are jeopardized as in patient with a coexistent ulnar neuropathy. Technically, the position of the thumb can alter distance measurement. The thumb should be kept slightly extended but relaxed enough to eliminate muscular artifact that affects the recording of radial SNAP. Of special note is that the superficial radial sensory nerve has been reported to be less affected than the median nerve in demyelinating neuropathies;^20^ in such situations, appropriate treatment of a demyelinating neuropathy may be hindered by falsely diagnosing CTS based on the thumdiff study.

In sensory NCS, co-stimulation of the median and ulnar nerves is not usually a problem except when recording D4, which has dual sensory supply from both nerves. For example, the finding of an absent or prolonged D2 median SNAP latency and a normal D4 median SNAP latency is a red flag for co-stimulation of the ulnar nerve when recording D4 median SNAP. When co-stimulation occurs, the median and ulnar SNAPs recorded from D4 are usually identical in amplitudes and latencies. In sensory studies, high-stimulus intensity can provoke volume-conducted motor potentials that may obscure the sensory potentials; this is more often seen when recording D5 ulnar SNAP.

When sensory or motor potentials are unrecordable, one should ensure that the machine settings (pulse duration, high- and low-frequency filters) are accurate and the electrodes are properly connected to the channel amplifier, which should be turned on.

## Short segments studies

**Figure 4:**
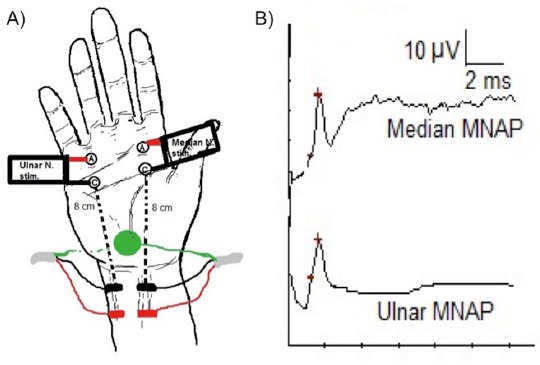
Palmar orthodromic mixed nerve study. **A)** Electrodes placement (black: active, red: reference, and green: ground) and stimulation sites. **B)** The recorded median and ulnar mixed nerve action potential (MNAP) waveforms and peak latency comparison.

**Figure 5:**
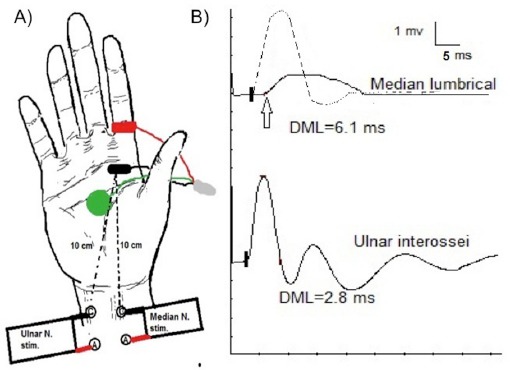
Lumbrical-interossei comparison study. **A)** Electrodes placement (black: active, red: reference, and green: ground) and stimulation sites. **B)** In CTS, the median distal motor latency is prolonged (6.1 ms, arrow) in comparison to the ulnar latency (2.8 ms). The dashed-line in the top trace shows the change in median waveform morphology when ulnar co-stimulation occurs.

The use of short segments studies has further improved the diagnostic sensitivity of NCS. In these studies, conduction velocity is computed for the area of pathology included in the short segment of the median nerve across the carpal tunnel, thereby excluding the dilution effect of the normal segment of the median nerve distal to the carpal tunnel.^1^,^10^ These studies include the palmar orthodromic mixed nerve study (**Figure 4A**), segmental median sensory CV across the wrist (**Figure 3B**), and lumbrical-interossei latency comparison study (**Figure 5A**).

## Palmar orthodromic mixed nerve study

The median and ulnar nerves are recorded by placing the A electrode just proximal to the wrist over the anatomic site for the median and ulnar nerves, respectively. The respective R electrode is placed 4 cm proximal to the A electrode, and the G electrode is placed on the dorsum of the hand. The median nerve is stimulated in the palm over the web space between D2 and D3 while the ulnar nerve is stimulated over the web space between D4 and D5. The distance between the A electrode and the cathode is 8 cm for both nerves (**Figure 4A**). The recorded median mixed nerve action potential (MNAP) has both motor and sensory components that are evoked by the stimulation of the motor fibers innervating the lumbricals and the afferent sensory fibers from D2 and D3.^21^ However, the recorded potential at the wrist is generated primarily from the sensory fibers.^22^ A relative prolongation of the median MNAP PL by ≥ 0.3 ms in comparison to the ulnar MNAP is considered abnormal.^19^ Because of the short distance used in this study, a minor error in the measurement can cause a significant change in the results. Stimulus shock artifact is sometimes troublesome especially with ulnar stimulation; using lower-stimulus intensities and rotating the anode around the cathode are helpful techniques to mitigate this problem.

## Segmental median sensory CV across the wrist

Using a technique similar to the one implemented for D2 median SNAP recording, the median nerve is stimulated at the wrist at 14 cm from the A electrode, and in the palm at 7 cm from the A electrode (**Figure 3B**). In CTS, conduction velocity of wrist-to-palm segment is slower than palm-to-digit by >10 m/s.^23^ Conduction block is identified when SNAP amplitude drops by ≥50% with stimulation at the wrist compared to stimulation at the palm. This technique is important for detection of sensory conduction block, which can be missed as axonal loss by other techniques that do not involve palm stimulation. The segmental measurement of CV can sometimes help distinguish CTS from peripheral neuropathy. In the latter, the maximal CV slowing occurs at palm-to-digit segment rather than across the wrist as in CTS.

## Lumbrical-Interossei latency comparison study

The A electrode is placed over the motor endplate which is usually localized at 1 cm proximal and lateral to the midpoint of the third metacarpal.^24^ The R electrode is placed over the metacarpal-phalangeal joint of D2. The median and ulnar nerves are stimulated at the wrist, 10 cm proximal to the A electrode (**Figure 5A**). The test is considered abnormal when the median distal motor latency (DML) is prolonged >0.5 ms relative to the ulnar.^24^ The position of the A electrode allows recording from both the second lumbrical and the first palmar interosseous depending on whether stimulation was delivered to the median or the ulnar nerve, respectively. Thus, inadvertent co-stimulation of both nerves provokes a CMAP co-recorded from both muscles simultaneously, and is recognized by observing an abrupt change in the median waveform morphology once ulnar nerve co-stimulation occurs (**Figure 5B**). The optimal placement of the A electrode is extremely important in obtaining valid and reliable results. The motor potential recorded from the motor endplate yields an initial negative deflection with an abrupt rise-time and high amplitude.^24^ If the recorded potential does not have the aforementioned features, the A electrode should be repositioned until a satisfactory recording from the motor endplate is obtained. It is important to note that if the A electrode is not optimally placed, the recorded latency will not be interpretable using the commonly used cuff-off values.^24^

The median nerve axons supplying the second lumbrical are relatively spared compared with those supplying the APB even in patients with a late-stage CTS.^25^ In these cases, the study is useful in demonstrating a prolonged DML to the second lumbrical, even when the routine median sensory and motor potentials are absent.^26^ The study is also helpful in demonstrating prolonged DML of the median nerve out of proportion to that of the ulnar nerve when CTS is superimposed on peripheral neuropathy.^26^,^27^

## The Combined Sensory Index (CSI)

The CSI has been proposed to increase the sensitivity and specificity of NCS for the confirmation of CTS. The CSI is the sum of latency differences of three comparison tests (palmdiff + ringdiff + thumbdiff). A CSI score ≥ 1.0 is considered abnormal, having a sensitivity of 83% and a specificity of 95%.^19^ However, an unequivocally high-latency difference in any one of the three tests (palm diff ≥ 0.4 ms, ring diff ≥ 0.5 ms, thumb diff >0.7 ms) confidently predicts an abnormal CSI without losing its specificity, negating the need to do all 3 tests.^28^

## Failure to achieve supramaximal stimulation:

A common pitfall is to stop increasing the stimulus intensity once the potential is within normal range. The “normal” submaximal potential does not represent the true potential that would be recorded when all nerve axons are stimulated rendering any concurrent or follow-up comparison study unreliable. It is impossible to ascertain stimulation of all nerve axons unless supramaximal stimulation is delivered. Submaximal stimulation at a proximal site may reveal a false impression of conduction block or slow CV caused by failure to stimulate all fast conducting axons. Conversely, one should avoid starting with a high-stimulation pulse because of the following: 1) it is painful; 2) a supramaximal response can often be obtained with less stimulus intensity; and 3) such technique deprives observing the growth of the amplitude and the change in the waveform morphology, impairing recognition of stimulus lead and co-stimulation as discussed below.

## Co-stimulation and stimulus lead

**Figure 6:**
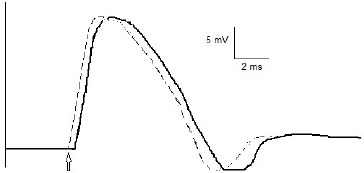
Stimulus lead. Median compound muscle action potential (CMAP) waveform is shown. Note the shortening of distal motor latency without increment in the amplitude (dashed-line) indicating a stimulus lead.

The downside of overstimulation is the possibility of co-stimulating the ulnar nerve during median nerve stimulation. Fortunately, it is not a common problem when appropriate stimulation techniques are implemented. Co-stimulation is usually encountered when unexpectedly high stimulus intensity is required to achieve supramaximal response, as in obese subjects, or in slim subjects in whom the appropriate supramaximal stimulation is high enough to co-stimulate the ulnar nerve. Features that help recognize ulnar nerve co-stimulation include: 1) a change in the median waveform morphology from a doom to a bifid shape during stimulus increment (**Figure 1B**); 2) the presence of an initial positive deflection from the baseline indicating volume conduction from ulnar-innervated thenar muscles; 3) an unexpectedly high median CMAP amplitude; and 4) contraction of both median- and ulnar-innervated hand muscles during median nerve stimulation. Once co-stimulation is recognized, the stimulus intensity should be lowered gradually until the waveform reverts to its normal doom-shaped morphology. Stimulus lead occurs when overstimulation provokes a larger zone of depolarization beneath the cathode, which stimulates the nerve at a distance shorter than that measured superficially from the A electrode, causing a falsely shorter latency. A stimulus lead is readily recognized during stimulus intensity increment when a decrease in latency occurs without the accompanying growth in amplitude (**Figure 6**).

## Review the waveforms

Inspecting the morphology of the waveforms and the placement of their markers is essential to ensure the accuracy of the study. Although, the machine automatically places the markers for latencies, amplitude and duration, the electromyographer should recheck their placement to avoid erroneous results. Identification of the changes in the shape of the waveform helps recognize co-stimulation (discussed above). With distal median nerve stimulation, an initial positive deflection from the baseline indicates that the A electrode is not placed directly on the motor endplate of the APB. In addition, waveform inspection helps recognize anomalous innervation (discussed below), temporal dispersion and conduction block.

## Recognition of median-ulnar anastomosis

**Figure 7:**
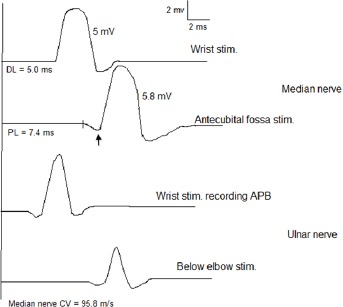
Combination of Martin-Gruber Anastomosis (MGA) and Carpal Tunnel Syndrome (CTS). An MGA with cross-over fibers innervating the thenar muscles. Routine median motor study reveals higher compound muscle action potential (CMAP) amplitude with antecubital-fossa stimulation than that obtained with wrist stimulation. Ulnar nerve stimulation recording thenar muscles shows a drop in CMAP amplitude with proximal stimulation. With a coexistent CTS, there is a positive deflection (arrow) with antecubital- fossa stimulation, and a factitiously fast conduction velocity of the median nerve in the forearm.

Anastomosis between the median and ulnar nerves is common,^29^ and can cause difficulties in interpreting NCS. Martin-Gruber Anastomosis (MGA) is a crossover of motor fibers from the median to the ulnar nerve in the forearm. The crossing fibers run distally with the ulnar nerve to innervate one or more of the following muscles: First Dorsal Interosseous (FDI), hypothenar or thenar muscles.^29^ When the crossover fibers innervate one of the ulnar-innervated thenar muscles, proximal median nerve stimulation provokes higher CMAP amplitude than that provoked with distal stimulation. This is because with proximal stimulation, above the crossover, the recorded potentials arise from both the median- and ulnar-innervated thenar muscles, whereas with distal stimulation, below the crossover, the recorded potentials arise only from the median-innervated thenar muscles. When CTS coexists with this type of MGA, the cross-over fibers that bypass the carpal tunnel through the ulnar nerve reach the thenar muscles faster than the median nerve fibers that are delayed at the carpal tunnel, leading to an initial positive deflection of the waveform with proximal-median nerve stimulation and a fast-conduction velocity in the forearm (**Figure 7**). Volume conduction from the thenar muscles is responsible for the initial positive deflection of the waveform. The presence of an MGA is confirmed by demonstrating a drop in thenar CMAP amplitude with ulnar nerve stimulation below the elbow as compared to stimulation at the wrist.^30^ Other variants of MGA are beyond the scope of this article.

**Figure 8:**
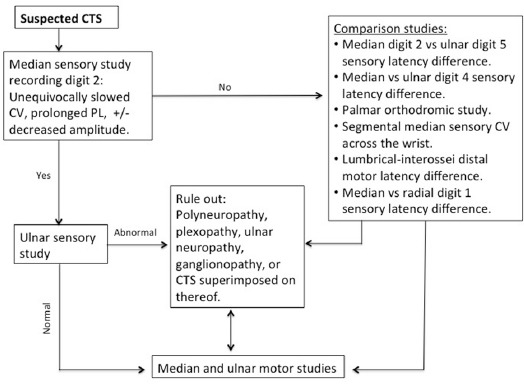
Nerve conduction study algorithm for patients with suspected CTS.

Riche-Cannieu Anastomosis (RCA) involves a communication between the deep branch of the ulnar nerve and the recurrent branch of the median nerve in the palm, leading to a complete or partial innervation of thenar muscles by the ulnar nerve. In normal subjects, this anomaly is electrophysiologically recognized when the median sensory response is normal but the motor response is absent^31^ or diminished^32^ despite a normal bulk and strength of the thenar muscles. An RCA is confirmed by finding a normal thenar CMAP with ulnar nerve stimulation at the wrist.^30^ When CTS coexists with an RCA, the NCS findings do not follow the expected pattern depicted in **Figure 8**. The median sensory latencies will be delayed or absent based upon CTS severity, whereas the APB CMAP is absent or diminished due to the RCA. Needle EMG of the APB can be normal when it is completely innervated by the ulnar nerve (through the communicating branch) or minimally abnormal when it is partially innervated by the median nerve. Another rarer anomaly is, when all motor and sensory innervation of the hand is provided by the ulnar nerve, “all-ulnar-hand”.^33^ Awareness of these anomalies is important for the accurate interpretation of the findings of the NCS in such cases.

## Median nerve CV

Slowing of the median nerve CV in the forearm is sometimes encountered in severe CTS.^10^ The possible explanations are that the presence of conduction block at the carpal tunnel and/or the occurrence of retrograde degeneration of the fastest conducting fibers proximal to the carpal tunnel will exclude these fibers form the contribution to the measured CMAP, leaving the slower conducting fibers as the main contributor to the calculation of CV.^1^

## When to do EMG?

The AANEM practice recommendations listed needle EMG as an optional procedure when evaluating patients with CTS.^8^ Needle EMG is useful in confirming axonal loss in CTS especially when it is not clear whether a drop in CMAP amplitude is due to distal conduction block or axonal loss. In addition, needle EMG is essential for the assessment of other confounding or coexistent conditions (**Table 1**).

## Incomplete study

One of the major pitfalls when evaluating a patient with suspected CTS is the early termination of the study after demonstrating a prolonged median distal latency and before performing the minimum electrodiagnostic tests recommended by the AANEM.^8^ It is not uncommon that a patient referred for CTS is found to have a coexistent or a completely different diagnosis. Therefore, the EDX should be tailored to the clinical assessment without jeopardizing the AANEM practice recommendations (**Figure 8**).

## Electrodiagnostic grading scale

**Figure 9:**
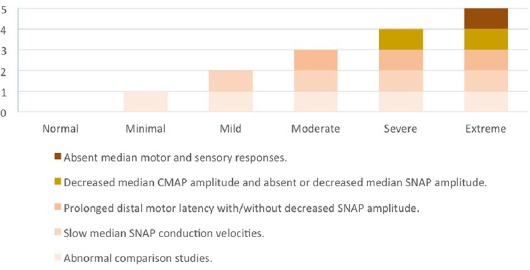
An NCS grading scale for the severity of CTS. The severity of involvement of NCS parameters generally follows the pattern depicted in the chart above. An unequivocal deviation from this pattern indicates a coexistent or a different diagnosis.

Grading the severity of CTS based on EDX findings has been debated in the literature.^34^,^35^ It is important to mention that the EDX grading scale is an objective measure for the severity of the “median neuropathy at the wrist” and that it does not measure the subjective severity of the clinical symptoms, which is based on patient report. The grading scale in this article (**Figure 9**) is derived from the previously reported scales.^1^,^36^,^37^ It is left to the discretion of the electromyographer whether or not to use a grading scale when reporting the findings of the EDX study. Nonetheless, it is important to understand the evolution of sensory and motor NCS findings with each EDX grade, that is, when moving to a higher NCS grade, the findings of the lower grades are usually satisfied (**Figure 9**). For example, when median DML is prolonged, the median sensory CV/PL is expected to be slow; and when median CMAP amplitude is decreased, the DML is expected to be prolonged and the sensory CV/PL is expected to be slow. As such, unequivocal deviation from this pattern is helpful in suggesting the presence of a superimposed or an alternative condition.

## Uncomplicated CTS (no superimposed conditions)

When approaching CTS, the author prefers to start with D2 median and D5 ulnar sensory NCS (**Figure 8**). The presence of unequivocal slowing of CV or prolongation of PL of D2 median SNAP, along with normal D5 ulnar SNAP CV and PL, is sufficient to demonstrate median neuropathy at the wrist. If D2 median CV and PL are normal or borderline slowed, a minimum of 2 abnormal comparison techniques are required to provide an electrophysiological evidence of median neuropathy at the wrist. At the author’s institute, the following comparison studies are routinely performed and their findings are considered abnormal when: 1) a prolonged D2 median versus D5 ulnar SNAP PL ≥ 0.5 ms; 2) a prolonged D4 median vs D4 ulnar SNAP PL ≥ 0.5 ms, and 3) a prolonged median vs. ulnar palmar MNAP PL≥0.3 ms. At least two abnormal comparison tests are required to lower the risk of false positive and false negative results.^9^ Next, median and ulnar motor NCS are performed. If the median SNAP is absent, a prolonged median DML will localize the lesion to the wrist, provided that the ulnar DML is normal. When both median sensory and motor responses are absent, the lumbrical-interossei comparison study should be conducted.^25^ For such uncomplicated CTS, NCS of the asymptomatic opposite hand is not routinely required.^8^

## Confounding factors of CTS

By confounders, we refer to the presence of coexistent conditions that contribute to the clinical presentation and/or the EDX findings such as ulnar neuropathy, cervical radiculopathy, etc. Meticulous history and neurological examination are essential to determine whether the symptoms are inconsistent with or not fully explained by CTS, generate differential diagnosis, and to help plan an EDX study. In these cases, the findings of NCS do not strictly follow the pattern depicted in **Figure 9**. For example, much better preservation of median SNAP amplitude in comparison to median CMAP amplitude may indicate a coexistent C8/T1 radiculopathy, MND, MMN, neurogenic thoracic outlet syndrome, distal myopathy, or rarely, recurrent median motor branch neuropathy. In severe CTS, when both median sensory and motor responses are absent, yet lumbrical-interossei study demonstrates slowing at the wrist; the finding of low ulnar CMAP amplitude with preserved ulnar SNAP is suggestive of a superimposed C8/T1 radiculopathy. In these cases, it is essential to perform needle EMG to help further localize the lesion and clarify the underlying etiology (**Table 1**).

In conclusion, EDX studies are frequently performed on daily basis for the evaluation of CTS. Integration of the medical history and neurological examination is important to generate differential diagnosis and to plan the EDX study accordingly. An EDX study can confirm the clinical diagnosis of CTS, assess its severity, and rule in/out coexistent conditions. There are many technical and physiological pitfalls and artifacts that should be recognized and rectified promptly to avoid misdiagnosis. When coexisting or confounding diagnoses are suspected, the electromyographer should extend the EDX study to clarify the underlying condition and achieve accurate diagnosis.
